# Impact of Gonadotropin-Releasing Hormone Agonist Pre-treatment on the Cumulative Live Birth Rate in Infertile Women With Adenomyosis Treated With IVF/ICSI: A Retrospective Cohort Study

**DOI:** 10.3389/fendo.2020.00318

**Published:** 2020-05-29

**Authors:** Minghui Chen, Lu Luo, Qiong Wang, Jun Gao, Yuqing Chen, Yingying Zhang, Canquan Zhou

**Affiliations:** ^1^Reproductive Medicine Center, the First Affiliated Hospital of Sun Yat-sen University, Guangzhou, China; ^2^Guangdong Provincial Key Laboratory of Reproductive Medicine, the First Affiliated Hospital of Sun Yat-sen University, Guangzhou, China; ^3^Department of Gynecology, the First Affiliated Hospital of Sun Yat-sen University, Guangzhou, China

**Keywords:** GnRH agonist, ovarian stimulation, adenomyosis, *in vitro* fertilization, live birth rate

## Abstract

**Introduction:** Although pre-treatment with a GnRH agonist can reduce the size of adenomyosis lesions, the supra-physiological hormone level induced by controlled ovarian hyperstimulation (COH) may negate the usefulness of the GnRH agonist in patients with adenomyosis lesions, leading to continued poor outcomes in fresh embryo transfer cycles during *in vitro* fertilization (IVF). It is unclear whether GnRH agonist pre-treatment before starting the long GnRH agonist protocol for IVF/ICSI (intracytoplasmic sperm injection) can improve cumulative live birth rate (CLBR) of infertile women with adenomyosis.

**Method:** In this retrospective cohort study, a total of 374 patients diagnosed as adenomyosis (477 cycles) underwent IVF/ICSI with long GnRH agonist protocol with or without GnRH agonist pre-treatment between January 2009 and June 2018. Logistic regression was used to assess the association between GnRH agonist pre-treatment and pregnancy outcome after adjusting for confounding factors.

**Results:** The live birth rate in fresh embryo transfer cycles was higher in the non-pre-treatment group than in the GnRH agonist pre-treatment group (37.7 vs. 21.2%, *P* = 0.028); the adjusted odds ratio (OR) for the long agonist protocol without pre-treatment was 1.966 (95% CI: 0.9–4.296, *P* = 0.09). The CLBR was higher in the non-pre-treatment group than in the GnRH agonist pre-treatment group (40.50 vs. 27.90%, *P* = 0.019); the adjusted OR for the long agonist protocol without pre-treatment was 1.361 (95% CI: 0.802–2.309, *P* = 0.254).

**Conclusion:** Our results indicated that GnRH agonist pre-treatment before starting the long GnRH agonist protocol does not improve the live birth rate in fresh embryo transfer cycles or CLBR in infertile women with adenomyosis after IVF/ICSI treatment when compared to that in non-pre-treated patients. A subsequent prospective randomized controlled study is needed to confirm these results.

## Introduction

Uterine adenomyosis is a common gynecological disorder characterized by the presence of ectopic endometrial glands and stroma surrounded by hyperplastic smooth muscle within the myometrium. Clinical manifestations include pelvic pain, abnormal uterine bleeding, and infertility. Results from two recent meta-analyses have revealed that adenomyosis has a detrimental effect on *in vitro* fertilization (IVF) outcomes, resulting in a reduced implantation rate, reduced pregnancy rate, reduced live birth rate, and an increase in miscarriage risk (1, 2).

Although the pathogenic mechanisms underlying the development of adenomyosis are unclear, it is well-understood that adenomyosis grows and declines in an estrogen-dependent manner. Adenomyotic tissue contains estrogen receptors (ER), progesterone, and androgen receptors. In addition, aromatase and sulfatase enzymes—which catalyze the conversion of androgens to estrogens—can be found in adenomyotic tissues. Together with circulating estrogens, locally produced estrogens stimulate the growth of tissue through interactions with the ER (3). Therapy with an agonist for GnRH decreases the expression of aromatase cytochrome P450 in the eutopic endometrium; this protein is overexpressed in women with adenomyosis (4). In addition, administration of a GnRH agonist leads to a hypo-estrogenic status by suppression of the hypothalamic-pituitary axis. Therefore, it is foreseeable that treatment with a GnRH agonist can reduce the size of adenomyosis lesions (3). Successful spontaneous pregnancies following treatment with a GnRH agonist in infertile women with adenomyosis have been reported (5–7). Recently, a retrospective study compared patients with and without long-term GnRH agonist pre-treatment before the preparation of the endometrium with hormone-replacement therapy (HRT). In this study, long-term pre-treatment with the GnRH agonist significantly improved the implantation rate, clinical pregnancy rate, and on-going pregnancy rate of patients with adenomyosis in frozen embryo transfer (FET) cycles (8).

Although pre-treatment with a GnRH agonist can reduce the size of adenomyosis lesions, the supra-physiological hormone level induced by controlled ovarian hyperstimulation (COH) may negate the usefulness of the GnRH agonist in patients with adenomyosis lesions, leading to continued poor outcomes in fresh embryo transfer cycles during IVF. A retrospective study compared fresh embryo transfer cycles with or without GnRH agonist pre-treatment and showed no group difference in the clinical pregnancy rates of patients with adenomyosis (9). With the increasing use of embryo freezing-thawing, the cumulative live birth rate (CLBR) has been suggested as a suitable mode of reporting the success of an IVF program, which incorporates both fresh and freeze-thawed embryo transfer (10). It is unknown whether GnRH agonist pre-treatment can improve the CLBR in patients with adenomyosis after *in vitro* fertilization treatment. To answer this question, we devised this retrospective study.

## Materials and Methods

### Patients Population

This is a retrospective, single-center cohort study. Our patient population consisted of women with ultrasound-diagnosed adenomyosis who underwent IVF or ICSI, using the long GnRH agonist protocol, both with and without pre-treatment with a GnRH agonist between January 2009 and June 2018 at the Reproductive Medicine Center of the First Affiliated Hospital of Sun Yat-sen University. The sonographic diagnosis criteria of adenomyosis included: heterogeneous myometrial area, globular asymmetric uterus, irregular cystic spaces, myometrial linear striations, poor definition of the endometrial myometrial junction, myometrial anterior posterior asymmetry, thickening of the anterior and posterior myometrial wall, and increased or decreased echogenicity (11). The diagnosis was made by a single doctor in condition that the patients were in non-menstrual period and did not receive hormone therapy within 3 months. Cycles involving oocyte donation, oocyte sharing, oocyte cryopreservation, and/or frozen oocyte thawing were excluded from the analysis.

### Controlled Ovarian Stimulation Protocol

The patients were allocated to the GnRH agonist pre-treatment group and non-pre-treatment group by doctors' preference. In the GnRH agonist pre-treatment group, GnRH agonist pre-treatment was initiated at the early follicle phase by administration of up to three injections of 3.75 mg of triptorelin acetate (Ipsen Pharma Biotech, France). The uterine anteroposterior diameter was measured 28 days after each injection and if it was more than 70 mm, injection of the same dose of GnRH agonist would be repeated up till the third injection. COH was induced using the long GnRH agonist protocol with a long-lasting formulation of triptorelin acetate depot (1.0–1.8 mg) or a daily dose (0.05–0.1 mg) of triptorelin acetate for pituitary downregulation. GnRH agonist administration for pituitary downregulation initiated 28 days after the last injection of 3.75 mg of triptorelin acetate in the GnRH agonist pre-treatment group and in the mid-luteal phase of the previous cycle in the non-pre-treatment group (12, 13). Gonadotropin stimulation with recombinant FSH (150-300 IU; Gonal-F, Merck Serono, Darmstadt, Germany) was initiated 14 days after GnRH agonist downregulation. The dose of recombinant FSH was determined on the basis of the patient's age, weight, and ovarian reserve, with or without human menopausal gonadotropin (hMG; Livzon, Zhuhai, China). Final oocyte maturation was induced by administering human chorionic gonadotropin (hCG, Ovidrel 250 mg; Merck Serono, Darmstadt, Germany) when at least one follicle ≥18 mm in diameter or two follicles ≥17 mm in diameter could be visualized on ultrasonography. Oocyte retrieval was performed 34–36 h after hCG administration. Fertilization was performed using either standard insemination or intracytoplasmic sperm injection (ICSI). Embryo transfer was performed on either day 3 or 5. No more than three embryos were transferred. An intramuscular injection of progesterone (40 mg/day) was administered as luteal support.

### Vitrification and Preparation of the FET Cycle

Supernumerary embryos were cryopreserved if they met the following criteria: day 3 embryos with at least six blastomeres and ≤20% fragmentation or day 5–6 blastocysts with at least expansion stage 3, inner cell mass score A or B, and trophectoderm score A or B (according to the Gardner grading system) (14). The protocols for FET included the natural cycle and the hormone replacement therapy (HRT) cycle with endometrial preparation with exogenous estrogen and progesterone, with or without GnRH agonist pre-treatment (8). FET was performed 2 months after failure of fresh embryo transfer.

### Outcome Measures

The primary outcome measure was the CLBR per ovarian stimulation cycle. The secondary outcome was the live birth rate per fresh embryo transfer cycle. A clinical pregnancy was defined as the presence of at least one intrauterine gestational sac, as visualized by ultrasonography. A miscarriage was defined as the loss of a clinical pregnancy before 12 weeks of gestation. A live birth was defined as any birth event in which at least one baby was born alive. The CLBRs were calculated by including the first live births generated during the IVF/ICSI cycles as the numerator and censoring additional live births. The denominator was defined as all ovarian stimulation cycles.

### Statistical Analysis

Continuous data were assessed for normality using the Shapiro–Wilk test, and the data were expressed as mean (±SD) or median (interquartile range), depending on the distribution. Categorical data were presented as frequency and percentage within each study group. Inter-group differences were assessed using Student's *t*/Mann–Whitney tests and chi-squared tests for continuous and categorical data, respectively. Fisher's exact test was applied when the expected values in any of the cells of a contingency table were below 5; in all other cases, Pearson's chi-squared test was applied. The association between GnRH agonist pre-treatment and pregnancy outcome was evaluated by multivariable logistic regression analysis while adjusting for potential confounders. Statistical significance was set at *P* ≤ 0.05. Analyses were performed using IBM SPSS statistics (version 25; IBM, Chicago, US).

## Results

### Study Population

**Figure 1:**
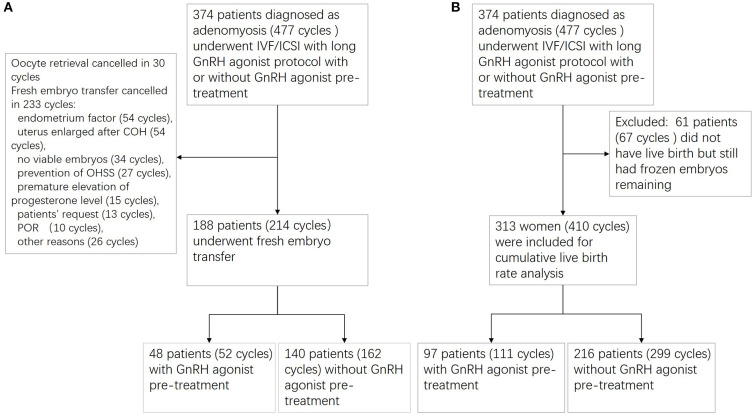
Flowchart of patient recruitment. **(A)** Flowchart for fresh embryo transfer cycles; **(B)** flowchart for cumulative live birth rate analysis.

Between January 2009 and June 2018, a total of 374 patients diagnosed as adenomyosis (477 cycles) underwent IVF/ICSI with long GnRH agonist protocol with or without GnRH agonist pre-treatment. Among the 374 patients, 313 adenomyosis patients (410 cycles) were included in the analysis of the CLBR and the other 61 patients (67 cycles) who did not have live birth but still had frozen embryos remaining were excluded. Ninety-seven patients (111 cycles) received GnRH agonist pre-treatment, whereas the remaining 216 patients (299 cycles) did not receive pre-treatment. Among the 374 patients, 188 patients (214 cycles)underwent fresh embryo transfer; 48 patients (52 cycles) in the pre-treatment group and 140 patients (162 cycles) in the non-pre-treatment group. Oocyte retrieval was canceled in 30 cycles; fresh embryo transfer was canceled in 233 cycles for the reasons included: endometrium factor (54 cycles), uterus enlarged after COH (54 cycles), no viable embryos (34 cycles), prevention of OHSS (27 cycles), premature elevation of progesterone level (15 cycles), patients' request (13 cycles), poor ovarian response (10 cycles), other reasons (26 cycles) (Figure 1).

### Baseline Characteristics and Treatment Characteristics in Fresh Embryo Transfer Cycles

In fresh embryo transfer cycles, LH level and progesterone level on day of hCG administration were higher in the non-pre-treatment group (*P* < 0.001 and *P* = 0.032). The number of oocytes retrieved, mature oocytes, oocytes fertilized, viable embryos, high-quality embryos were higher in the non-pre-treatment group (*P* = 0.007, 0.010, 0.001, 0.004, and 0.004). The remaining baseline characteristics and treatment characteristics were comparable between the two groups (Table 1).

**Table 1:** Patient characteristics and pregnancy outcomes in fresh embryo transfer cycles by stimulation protocol.

	**Non-pre-treatment group**	**GnRH agonist pre-treatment group**	***P*-value**
No. of cycles	162	52	
Female age (years)	33.5 (30–36.75)	33.5 (31–36.75)	0.727^b^
Male age (years)	36 (32–39)	36 (33–38)	0.838^b^
Duration of infertility (years)	4 (2–6)	4 (2–6.75)	0.851^b^
Body mass index (kg/m^2^)	20.80 (19.5–23.3)	20.55 (18.95–23.375)	0.637^b^
Complicated with endometriosis	63 (38.9%)	24 (46.2%)	0.353^c^
Type of infertility			0.266^c^
primary	76 (46.9%)	29 (55.8%)	
secondary	86 (53.1%)	23 (44.2%)	
Gravidity	1 (0–2)	0 (0–2)	0.511^b^
Parity	0 (0–0)	0 (0–0)	0.845^b^
Times of previous miscarriage	0 (0–0)	0 (0–0)	0.561^b^
Insemination method			0.915^d^
IVF	127 (78.4%)	42 (80.8%)	
ICSI	29 (17.9%)	8 (15.4%)	
IVF + ICSI	6 (3.7%)	2 (3.8%)	
Basal FSH (mIU/mL)	5.74 (4.88–6.74)	6.19 (4.99–7.39)	0.113^b^
Antral follicle count	9 (6–12)	7.5 (5–11.75)	0.053^b^
Endometrial thickness at mid-luteal phase	11.26 (10–13)	11.00 (9–12)	0.163^b^
Anteroposterior diameter of uterus at mid-luteal phase	55 (47–62)	55.97 (43–60)	0.612
Stimulation duration (day)	11 (10–12)	11 (10–12)	0.851^b^
Total dosage of gonadotropin (IU)	2,647.44 (801.886)	2,609.81 (742.989)	0.809^a^
LH level on day of hCG administration (mIU/mL)	0.77 (0.55–1.11)	0.38 (0.30–0.57)	<0.001^b^^*^
Estrogen level > 3,000 ng/mL on day of hCG administration	62 (38.3%)	11 (21.2%)	0.893^c^
Progesterone level on day of hCG administration (ng/mL)	0.6 (0.4–0.9)	0.5 (0.33–0.71)	0.032^b^^*^
Number of oocytes retrieved	10 (7–15)	8 (5.25–12)	0.007^b^^*^
Number of mature oocytes	9 (6–12)	7 (4–11)	0.010^b^^*^
Number of oocytes fertilized	6.5 (4–9)	4.5 (3–7)	0.001^b^^*^
Number of viable embryos	3.5 (2–6)	3 (2–4)	0.004^b^^*^
Number of high-quality embryos	3 (1–5)	2 (1–3)	0.004^b^^*^
Number of fresh embryos transferred	2 (2–3)	2 (2–3)	0.875^b^
Number of high-quality embryos transferred	2 (1–2)	2 (1–2)	0.674^b^
Type of embryo transferred			0.457^d^
Cleavage embryo	153 (94.4%)	51 (98.1%)	
blastocyst	9 (5.6%)	1 (1.9%)	
Clinical pregnancy	69 (42.6%)	16 (30.8%)	0.130^c^
Miscarriage	8 (11.6%)	5 (31.3%)	0.063^d^
Preterm labor	7 (11.5%)	0 (0.0%)	0.585^d^
Live birth	61 (37.7%)	11 (21.20%)	0.028^c^^*^

a *Two-sample t-test. Values are means (SD)*.

b *Two-sample Mann–Whitney test. Values are medians (interquartile range)*.

c *Pearson chi-squared test. Values are number (percentage)*.

d *Fisher exact probability test. Values are number (percentage)*.

* *Statistical significance*.

### Pregnancy Outcome in Fresh Embryo Transfer Cycles

The live birth rate in the fresh embryo transfer cycles was higher in the non-pre-treatment group than in the GnRH agonist pre-treatment group (37.7 vs. 21.2%, *P* = 0.028). The clinical pregnancy rate, miscarriage rate, and preterm labor rate were comparable in the two groups (Table 1). To identify potential confounders that may interfere with the association analysis between the GnRH agonist pre-treatment and live birth, we compared baseline and treatment characteristics between the live birth and non-live birth groups. There were significant differences in female age, endometrial thickness at mid-luteal phase, number of oocytes fertilized, viable embryos, high-quality embryos, and high-quality embryos transferred between the two groups (Table 2). The adjusted odds ratio (OR) for the long agonist protocol without pre-treatment was 1.966 (95% CI: 0.9–4.296, *P* = 0.09) after adjustment for potential confounders (Table 2).

**Table 2:** Patient characteristics by live birth or no live birth in fresh embryo transfer cycles.

	**Live birth**	**No live birth**	***P*-value**	**Adjusted ORs**	***P*-value**
	**(*n* = 72)**	**(*n* = 142)**			
Female age (years)	32.96 (3.847)	34.2 (4.579)	0.050^a^	0.94 (0.872–1.013)	0.105
Male age (years)	36.13 (5.604)	35.7 (4.976)	0.576^a^		
Duration of infertility (years)	4 (2–7)	4 (2–6)	0.752^b^		
Body mass index (kg/m^2^)	20.7 (19.5–23.3)	20.8 (19.25–23.35)	0.578^b^		
Complicated with endometriosis	30 (41.70%)	57 (40.10%)	0.830^c^		
Type of infertility			0.628^c^		
Primary	37 (51.40%)	68 (47.90%)			
Secondary	35 (48.60%)	74 (52.10%)			
Gravidity	0 (0–2)	1 (0–2)	0.828^b^		
Parity	0 (0–0)	0 (0–0)	0.17^b^		
Times of previous miscarriage	0 (0–0)	0 (0–0)	0.813^b^		
Insemination method					
IVF	57 (79.20%)	112 (78.90%)	0.962^c^		
ICSI	12 (16.70%)	25 (17.60%)			
IVF + ICSI	3 (4.20%)	5 (3.50%)			
Basal FSH (mIU/mL)	5.74 (4.6–6.98)	5.87 (4.955–6.985)	0.38^b^		
Antral follicle count	9 (6–13)	9 (6–11.5)	0.310^b^		
Endometrial thickness at mid-luteal phase	12 (10–13)	11 (9–12)	0.012^b^^*^	1.106 (0.987–1.24)	0.083
Anteroposterior diameter of uterus at mid-luteal phase	54 (46.75–59.25)	55.97 (46.25–62.75)	0.297^b^		
Stimulation protocol			0.028^c^^*^		
Non-pre-treatment	61 (84.70%)	101 (71.10%)		1.966 (0.9–4.296)	0.09
GnRH agonist pre-treatment	11 (15.30%)	41 (28.90%)		Reference	
Stimulation duration (day)	11 (10–12)	11 (10–12)	0.518^b^		
Total dosage of gonadotropin (IU)	2700 (2000–3300)	2600 (2075–3062.5)	0.714^b^		
LH level on day of hCG administration (mIU/mL)	0.77 (0.51–1.13)	0.64 (0.4–0.96)	0.056^b^		
Estrogen level > 3,000 ng/mL on day of hCG administration	25 (34.70%)	48 (33.80%)	0.893^c^		
Progesterone level on day of hCG administration (ng/mL)	0.6 (0.4–0.8)	0.6 (0.4–0.9)	0.428^b^		
Number of oocytes retrieved	10 (8–15)	9 (6–13.5)	0.092^b^		
Number of mature oocytes	9 (6–12)	8 (6–11)	0.082^b^		
Number of oocytes fertilized	7 (5–9)	5 (3–8.5)	0.009^b^^*^	1.052 (0.937–1.18)	0.389
Number of viable embryos	4 (3–6)	3 (2–5.5)	0.024^b^^*^	0.915 (0.731–1.146)	0.441
Number of high-quality embryos	3 (2–5)	2 (1–4)	0.007^b^^*^	1.1 (0.846–1.429)	0.477
Number of fresh embryos transferred	2 (2-3)	2 (2–3)	0.441^b^		
Number of high-quality embryos transferred	2 (1–2)	2 (1–2)	0.011^b^^*^	1.485 (0.974–2.262)	0.066
Type of embryo transferred			0.736^d^		
Cleavage embryo	68 (94.40%)	136 (95.80%)			
blastocyst	4 (5.60%)	6 (4.20%)			

a *Two-sample t-test. Values are means (SD)*.

b *Two-sample Mann–Whitney test. Values are medians (interquartile range)*.

c *Pearson chi-squared test. Values are number (percentage)*.

d *Fisher exact probability test. Values are number (percentage)*.

* *Statistical significance*.

### Baseline Characteristics and Treatment Characteristics in CLBR Analysis

In the analysis of CLBR, the proportion of patients complicated with endometriosis and the proportion of primary infertility were lower in the non-pre-treatment group (42.10 vs. 53.20%, *P* = 0.046; 42.80 vs. 56.80%, *P* = 0.012). The antral follicle count was higher in the non-pre-treatment group (*P* = 0.039). The number of oocytes retrieved, mature oocytes, oocytes fertilized normally, viable embryos, and high-quality embryos were higher in the non-pre-treatment group (*P* = 0.003, 0.005, 0.005, 0.019, and 0.019). The remaining baseline characteristics and treatment characteristics were comparable between the two groups (Table 3).

**Table 3:** Patient characteristics and pregnancy outcomes by stimulation protocol for cumulative live birth rate analysis.

	**Non-pre-treatment group**	**GnRH agonist pre-treatment group**	***P*-value**
No. of cycles	299	111	
Female age (years)	34 (31–37)	34 (31–37)	0.895^a^
Male age (years)	36 (32–40)	37 (33–39)	0.393^a^
Duration of infertility (years)	4 (2–6)	4 (2–6)	0.715^a^
Body mass index (kg/m^2^)	21 (19.5–23.3)	21.3 (19.28–23.11)	0.715^a^
Complicated with endometriosis	126 (42.10%)	59 (53.20%)	0.046^b^^*^
Type of infertility			0.012^b^^*^
Primary	128 (42.80%)	63 (56.80%)	
Secondary	171 (57.20%)	48 (43.20%)	
Gravidity	1 (0–2)	0 (0–2)	
Parity	0 (0–0)	0 (0–0)	
Times of previous miscarriage	0 (0–0)	0 (0–0)	
Insemination method			0.716^b^
IVF	240 (80.30%)	93 (83.80%)	
ICSI	53 (17.70%)	16 (14.40%)	
IVF + ICSI	6 (2.00%)	2 (1.80%)	
Basal FSH (mIU/mL)	5.69 (4.76–7.02)	5.73 (4.76–7.11)	0.994^a^
Antral follicle count	8 (5–12)	7 (4–10)	0.039^a^^*^
Endometrial thickness at mid-luteal phase	11.26 (9–13)	11 (9–12)	0.052^a^
Anteroposterior diameter of uterus at mid-luteal phase	55.97 (49–62)	55.97 (45–60)	0.226^a^
Cycles canceled			0.722^b^
Cancel of oocyte retrieval	22 (7.40%)	8 (7.20%)	
Cancel of fresh embryo transfer	127 (42.50%)	52 (46.80%)	
Number of oocytes retrieved	9 (5–15)	7 (3–12)	0.003^a^^*^
Number of mature oocytes	8 (4–13)	6 (3–11)	0.005^a^^*^
Number of oocytes fertilized normally	6 (3–9)	4 (2–7)	0.005^a^^*^
Number of viable embryos	3 (2–6)	2.5 (1–4)	0.019^a^^*^
Number of high quality embryos	2 (0–5)	1 (0–3)	0.019^a^^*^
Cycles with supernumerary embryos	206 (68.9%)	70 (63.1%)	0.263^b^
Cumulative Live birth	121 (40.50%)	31 (27.90%)	0.019^a^^*^

a *Two-sample Mann–Whitney test. Values are medians (interquartile range)*.

b *Pearson chi-squared test. Values are number (percentage)*.

* *Statistical significance*.

### Cumulative Live Birth and COH Protocol

The CLBR was significantly higher in the non-pre-treatment group than in the GnRH agonist pre-treatment group (40.50 vs. 27.90%, *P* = 0.019, Table 3). To identify potential confounders that may interfere with the association analysis between the GnRH agonist pre-treatment and cumulative live birth, we compared baseline and treatment characteristics between the cumulative and non-cumulative live birth groups. There were significant differences in female and male ages, proportion of patients complicated with endometriosis, basal FSH, antral follicle counts, endometrial thickness at mid-luteal phase, number of oocytes retrieved, mature oocytes, oocytes fertilized normally, viable embryos, and high-quality embryos between the two groups (Table 4). The adjusted odds ratio (OR) for the long agonist protocol without pre-treatment was 1.361 (95% CI: 0.802–2.309, *P* = 0.254) after adjustment for potential confounders (Table 4).

**Table 4:** Patient characteristics by pregnancy outcome for cumulative live birth rate analysis.

	**No cumulative live birth**	**Cumulative live birth**	***P*-value**	**Adjusted ORs (95% CI)**	***P*-value**
	**(*n* = 258)**	**(*n* = 152)**			
Female age (years)	35 (32–38)	33 (30–36)	<0.001^a^^*^	0.897 (0.831–0.968)	0.005^*^
Male age (years)	37 (32.75–40)	35 (32–39)	0.029^a^^*^	1.024 (0.964–1.088)	0.434
Duration of infertility (years)	4 (2–6)	4 (2–6)	0.841^a^		
Body mass index (kg/m^2^)	21.21 (19.5–23.3)	20.76 (19.4–23.05)	0.11^a^		
Complicated with endometriosis	129 (50.00%)	56 (36.80%)	0.010^b^^*^	1.584 (0.986–2.545)	0.057
Type of infertility			0.807^b^		
Primary	119 (46.10%)	72 (47.40%)			
Secondary	139 (53.90%)	80 (52.60%)			
Gravidity	1 (0–2)	1 (0–2)	0.637^a^		
Parity	0 (0–0)	0 (0–0)	0.704^a^		
Times of previous miscarriage	0 (0–0)	0 (0–0)	0.445^a^		
Insemination method			0.580^b^		
IVF	213 (82.60%)	120 (78.90%)			
ICSI	41 (15.90%)	28 (18.40%)			
IVF + ICSI	4 (1.60%)	4 (2.60%)			
Basal FSH (mIU/mL)	5.91 (4.88–7.36)	5.49 (4.71–6.67)	0.007^a^^*^	0.897 (0.8–1.006)	0.064
Antral follicle count	7 (4–10)	9 (6–13)	<0.001^a^^*^	0.996 (0.938–1.057)	0.891
Endometrial thickness at mid-luteal phase	11 (9–12)	11.26 (10–13)	<0.001^a^^*^	1.098 (1.007–1.196)	0.034^*^
Anteroposterior diameter of uterus at mid-luteal phase	55.97 (48–60.25)	55.97 (48–62)	0.891^a^		
Stimulation protocol			0.019^b^^*^		
Non-pre-treatment	178 (69.00%)	121 (79.60%)		1.361 (0.802–2.309)	0.254
GnRH agonist pre-treatment	80 (31.00%)	31 (20.40%)		Reference	
Number of oocytes retrieved	6 (3–12)	11 (8–17)	<0.001^a^^*^	0.962 (0.848–1.09)	0.539
Number of mature oocytes	6 (2–11)	10 (7–15)	<0.001^a^^*^	0.993 (0.834–1.183)	0.938
Number of oocytes fertilized	4 (1–7)	7 (5–11)	<0.001^a^^*^	1.043 (0.9–1.21)	0.574
Number of viable embryos	2 (1–3)	5 (3–7)	<0.001^a^^*^	1.149 (0.977–1.352)	0.093
Number of high-quality embryos	1 (0–3)	3 (2–5)	<0.001^a^^*^	1.213 (1.015–1.449)	0.033^*^

a *Two-sample Mann–Whitney test. Values are medians (interquartile range)*.

b *Pearson chi-squared test. Values are number (percentage)*.

* *Statistical significance*.

## Discussion

To date, no studies have elucidated whether GnRH agonist pre-treatment is beneficial in improving the CLBR in patients with adenomyosis. In this study, our data showed that the live birth rate in fresh embryo transfer cycles and CLBR of infertile women with adenomyosis after IVF/ICSI treatment is higher among patients undergoing the GnRH long agonist protocol without GnRH agonist pre-treatment than in the group with pre-treatment. However, after adjustment for confounding factors such as female ages, antral follicle counts, endometrial thickness, number of oocytes retrieved, mature oocytes, oocytes fertilized normally, viable embryos, and high-quality embryos, we show that GnRH agonist pre-treatment status is not associated with the live births or cumulative live births. A previous retrospective study showed GnRH agonist pre-treatment did not improve the clinical pregnancy rate of women with adenomyosis after fresh embryo transfer (9). Our results indicate that GnRH agonist pre-treatment also does not improve the live birth rate in fresh embryo transfer cycles or the CLBR of women with adenomyosis after IVF/ICSI.

Evidence from a systematic review suggests that administration of a GnRH agonist for 3–6 months before IVF or ICSI in women with endometriosis increases the odds of clinical pregnancy four-fold; this analysis included randomized controlled trials using any GnRH agonist before IVF or ICSI to treat women with any degree of endometriosis diagnosed by laparoscopy or laparotomy (15). Although adenomyosis and endometriosis share many diagnostic, symptomatic, and molecular similarities, the two conditions are distinct entities—many differences have been observed in their pathogenesis, risk factors, and clinical presentation (16). These differences may explain the differential impact of GnRH agonist pre-treatment on pregnancy outcome in IVF for patients with adenomyosis vs. endometriosis.

A recent retrospective study identified that long-term GnRH agonist pre-treatment significantly improved the implantation rate, clinical pregnancy rate, and on-going pregnancy rate of patients with adenomyosis in FET cycles (8). The authors suggested that this may have resulted from the observation that the GnRH agonist can induce a hypo-estrogenic effect by suppressing the hypothalamus–pituitary–ovary axis with a resultant reduction in adenomyosis and subsequent symptomatic relief (8). Moreover, exogenous treatment with a GnRH agonist significantly suppressed the proliferation of cells derived from the endometrium and the expansion of pathologic lesions in patients with adenomyosis (17). Nevertheless, COH following GnRH agonist pre-treatment will undoubtedly result in a supra-physiological elevation of estrogen levels, leading to re-enlargement of the uterus; therefore, this may offset the expected uterine shrinkage with GnRH agonist pre-treatment. Our results show that after adjustment for confounding factors, GnRH agonist pre-treatment status is not associated with live births.

A prospective study of 74 infertile patients with surgically proven endometriosis showed no significant differences in the implantation rate, miscarriage rate, and clinical pregnancy rate following IVF/ICSI between women with and those without adenomyosis (18). Our results showed that there was no significant difference in proportion of adenomyosis with endometriosis between live birth group and no live birth group in fresh embryo transfer cycles. Besides, adenomyosis with endometriosis was not associated with cumulative live birth after adjustment for confounding factors. In addition, female age, endometrial thickness, and the number of high-quality embryos were associated with the cumulative live birth of women with adenomyosis treated with IVF/ICSI—this finding is consistent with results of previous research (19–22).

Our study has notable strengths. It is the first study that has demonstrated the impact of GnRH agonist pre-treatment on the CLBR of infertile patients with adenomyosis after IVF treatment. Furthermore, the association between ovarian stimulation protocols and cumulative live births was evaluated by multivariable logistic regression models with adjustment for potential confounders. Our study has some limits. Firstly, it is a retrospective cohort study and therefore selection bias may exist. The patients were allocated to the GnRH agonist pre-treatment group and non-pre-treatment group by doctors' preference. Even so, the most baseline characteristics in the two groups are comparable. Although the complete data of indicators for severity of adenomyosis as uterus volume or serum CA125 were not available, anteroposterior diameters which can partly reflect the uterus volume were comparable between the two groups. Secondly, information of severity of the disease is lacking. However, at present there is no consistent standard for grading the severity of adenomyosis. Thirdly, data of cycle regimen for frozen-thawed embryo transfer was not analyzed. A meta analysis showed that there was no evidence of a difference between natural FET cycle and HRT FET cycle in the clinical pregnancy rate or a difference between natural FET cycle and HRT plus GnRH agonist suppression FET cycle in live birth rate (23). Therefore, the association analysis between GnRH agonist pre-treatment and pregnancy outcome may not be interfered by FET regimen status. Above all, a subsequent prospective randomized controlled study is needed to confirm our results in the future.

In conclusion, our results indicated that GnRH agonist pre-treatment before the long agonist protocol does not improve the live birth rate in fresh embryo transfer cycles or CLBR after IVF/ICSI among infertile women with adenomyosis.

## Data Availability Statement

The raw data supporting the conclusions of this manuscript will be made available by the authors, without undue reservation, to any qualified researcher.

## Ethics Statement

The studies involving human participants were reviewed and approved by the study was approved by the Institutional Ethics Committee from the First Affiliated Hospital of Sun Yat-sen University. Owing to the retrospective nature of the study, written informed consent was not required from the participants. Written informed consent for participation was not required for this study in accordance with the national legislation and the institutional requirements.

## Author Contributions

MC and LL contributed equally to design of the study, acquisition, analysis and interpretation of data, drafting, and revising the manuscript, and provided final approval of the manuscript prior to submission. QW contributed to the conception and design of the study, revising the manuscript, and provided final approval of the manuscript prior to submission. JG, YC, and YZ were involved in data acquisition and provided final approval of the manuscript prior to submission. CZ contributed to the conception and design of the study and provided final approval of the manuscript prior to submission.

## Conflict of Interest

The authors declare that the research was conducted in the absence of any commercial or financial relationships that could be construed as a potential conflict of interest.
